# Supplementary material for: The importance of the interface for picosecond spin pumping in antiferromagnet-heavy metal heterostructures
Source: Nat Commun. 2023 Feb 1;14:538. doi: 10.1038/s41467-023-36166-z (PMC9892507; doi:10.1038/s41467-023-36166-z)
Supplement: Supplementary file 1 — Supplementary Information [file 41467_2023_36166_MOESM1_ESM.pdf]

Supplementary Information
The importance of the interface for picosecond spin pumping in antiferromagnet-heavy metal heterostructures

Farhan Nur Kholid,[1] Dominik Hamara,[1] Ahmad Faisal Bin Hamdan,[1] Guillermo Nava Antonio,[1] Richard Bowen,[1] Dorothée Petit,[1] Russell Cowburn,[1] R.V Pisarev,[2] Davide Bossini,[3] Joseph Barker,[4] and Chiara Ciccarelli[1]

[1]*Cavendish Laboratory, University of Cambridge, Cambridge, CB3 0HE, United Kingdom*

[2]*Ioffe Physical-Technical Institute, Ferroics Physics Laboratory, Russian Academy of Sciences, St Petersburg, 194021, Russia*

[3]*Department of Physics, University of Konstanz, Universitaetsstrasse 10 78464 Konstanz, Germany*

[4]*School of Physics and Astronomy, University of Leeds, Leeds LS2 9JT, United Kingdom*

I. SD COUPLING HAMILTONIAN

We consider an antiferromagnetic insulator coupled to a Pt metal layer. We assume both have a cubic lattice. The AFM/NM interface normal is $\hat{z}$. The applied magnetic field and uniaxial anisotropy are in the plane along $\hat{x}$, so that if the field is not too big, the AFM spins are aligned along the $\pm\hat{x}$ direction. Thus we write the Hamiltonian of the antiferromagnet as

$$\mathscr{H}_{\mathrm{AFM}} = J_{\mathrm{AFM}} \sum_{i,j} \mathbf{S}_j \cdot \mathbf{S}_j + H \sum_i S_i^x - K \sum_i (S_i^x)^2 \tag{1}$$

where J_{AFM} is the exchange interaction within the AFM, H is the magnitude of an applied field along the x direction and K is a uniaxial anisotropy, noting that for small deviations from the energy minimum a cubic anisotropy is approximately uniaxial.

The electron Hamiltonian is a spin independent Hubbard model

$$\mathscr{H}_{\mathrm{Pt}} = \sum_{i,j} \left(t_{ij} c_i^\dagger c_j + \mathrm{h.c.} \right) \tag{2}$$

where t_{ij} is the hopping energy, $c_i^\dagger$ creates an electron at site i and c_j annihilates an electron at site j. This can be diagonalised giving

$$\mathscr{H}_{\mathrm{Pt}} = \sum_{\mathbf{k}} \varepsilon_{\mathbf{k}} c_{\mathbf{k}}^\dagger c_{\mathbf{k}} \tag{3}$$

where $\varepsilon_{\mathbf{k}}$ is the energy dispersion.

The coupling between the AFM spins and the Pt electrons is via an sd-coupling term. We ignore any distance dependence of the exchange and use a single parameter (J_{sd}) for the coupling of AFM spins and Pt electrons

$$\mathscr{H}_{sd} = \sum_i J_{sd} \mathbf{S}_i \cdot c_i^\dagger \boldsymbol{\sigma} c_i \tag{4}$$

where $\boldsymbol{\sigma} = (\sigma_x, \sigma_y, \sigma_z)$ is a vector of Pauli matrices. This Hamiltonian can be Fourier transformed into

$$\mathscr{H}_{sd} = \sum_{\mathbf{k},\mathbf{q}} J_{sd} \mathbf{S}_{\mathbf{q}} \cdot c_{\mathbf{k}}^\dagger \boldsymbol{\sigma} c_{\mathbf{k}+\mathbf{q}} \tag{5}$$

We specify the ground state spin configuration of the AFM. In general this can be written as

$$\mathbf{S}_i = S \mathrm{e}^{\mathrm{i}(\mathbf{K}_1+\mathbf{K}_2+\mathbf{K}_3)\cdot\mathbf{R}_i/2} \begin{pmatrix} 1 \\ 0 \\ 0 \end{pmatrix} \tag{6}$$

where $\mathbf{R}_i$ is the position of spin i and $\mathbf{K}_{1,2,3}$ are reciprocal lattice vectors which specify the ordering of the ground state and satisfy $\mathbf{K}_a \cdot \mathbf{R}_i = 2\pi n$ for integer n. For the $KNiF_3$ and $KCoF_3$ the G-type ground state is created using $\mathbf{Q} = (\mathbf{K}_1 + \mathbf{K}_2 + \mathbf{K}_3)/2$.

Under a small perturbation the transverse spin components (S_y, S_z) are

$$S_i^{y,z} = \sum_{\mathbf{q}} S_{\mathbf{q}}^{y,z} \mathrm{e}^{\mathrm{i}\mathbf{q}\cdot\mathbf{R}_i} \tag{7}$$

and expanding $S_i^x = \sqrt{S^2 - (S_i^y)^2 - (S_i^z)^2} \approx S - ((S_i^y)^2 + (S_i^z)^2)/2S$ for small S_i^y and S_i^z to second order we get

$$S_i^x \approx \mathrm{e}^{\mathrm{i}\mathbf{Q}\cdot\mathbf{R}_i} \left(S - \frac{(S_i^y)^2 + (S_i^z)^2}{2S} \right) \tag{8}$$

The Fourier transform is:

$$S_{\mathbf{q}}^x = S\delta_{\mathbf{qQ}} - \sum_{\mathbf{k}} \frac{S_{\mathbf{k}}^y S_{\mathbf{q}-\mathbf{Q}-\mathbf{k}}^y + S_{\mathbf{k}}^z S_{\mathbf{q}-\mathbf{Q}-\mathbf{k}}^z}{2S} \tag{9}$$

The exchange interaction within the AFM is written

$$\sum_{i\neq j} S_i^{y,z} S_j^{y,z} = \sum_{\mathbf{q}} \gamma_{\mathbf{q}} S_{\mathbf{q}}^{y,z} S_{-\mathbf{q}}^{y,z} \tag{10}$$

with a $\gamma_{\mathbf{q}} = \sum_{\boldsymbol{\delta}} \mathrm{e}^{\mathrm{i}\mathbf{q}\cdot\boldsymbol{\delta}}$ where $\boldsymbol{\delta}$ are the interaction vectors between sites, in this case the six nearest neighbour AFM interactions between the two sublattices.

The x-component is

$$\sum_{i\neq j} S_i^x S_j^x = \sum_{\mathbf{q}} \gamma_{\mathbf{q}} S_{\mathbf{q}}^x S_{-\mathbf{q}}^x \tag{11}$$

$$\approx -S \sum_{\mathbf{q}} \gamma_{\mathbf{q}} \delta_{\mathbf{qQ}} \sum_{\mathbf{k}} \frac{S_{\mathbf{k}}^y S_{-\mathbf{q}-\mathbf{Q}-\mathbf{k}}^y + S_{\mathbf{k}}^z S_{-\mathbf{q}-\mathbf{Q}-\mathbf{k}}^z}{2S} - S \sum_{\mathbf{q}} \gamma_{\mathbf{q}} \delta_{-\mathbf{qQ}} \sum_{\mathbf{k}} \frac{S_{\mathbf{k}}^y S_{\mathbf{q}-\mathbf{Q}-\mathbf{k}}^y + S_{\mathbf{k}}^z S_{\mathbf{q}-\mathbf{Q}-\mathbf{k}}^z}{2S} \tag{12}$$

$$= -\sum_{\mathbf{k}} \gamma_{\mathbf{Q}} \left(S_{\mathbf{k}}^y S_{-\mathbf{k}}^y + S_{\mathbf{k}}^z S_{-\mathbf{k}}^z \right) \tag{13}$$

On the second line only quadratic terms have been kept from the multiplication on the first line. We also used the fact that $2\mathbf{Q}$ is a reciprocal lattice vector.

The anisotropy term is basically the same except $\gamma_{\mathbf{Q}}$ is 1 (because it's an on-site interaction). The external field (again to second order in S) is

$$\sum_i S_i^x \approx -\sum_{\mathbf{k}} \frac{S_{\mathbf{k}-\mathbf{Q}}^y S_{-\mathbf{k}}^y + S_{\mathbf{k}-\mathbf{Q}}^z S_{-\mathbf{k}}^z}{2S} \tag{14}$$

All together we get the linearised AFM hamiltonian

$$\mathscr{H}_{\mathrm{AFM}} = \sum_{\mathbf{k}} \left(J_{\mathrm{AFM}}(\gamma_{\mathbf{k}} - \gamma_{\mathbf{Q}}) + K \right) \left(S_{\mathbf{k}}^y S_{-\mathbf{k}}^y + S_{\mathbf{k}}^z S_{-\mathbf{k}}^z \right) - \frac{H}{2S} \sum_{\mathbf{k}} \left(S_{\mathbf{k}-\mathbf{Q}}^y S_{-\mathbf{k}}^y + S_{\mathbf{k}-\mathbf{Q}}^z S_{-\mathbf{k}}^z \right) \tag{15}$$

We now write the sd Hamiltonian. Here we retain only the zeroth and first order terms (with respect to AFM spin operators) which are:

$$\mathscr{H}_{sd}^{(0)} = J_{sd} S \sum_{\mathbf{k}} c_{\mathbf{k}}^{\dagger} \sigma_3 c_{\mathbf{k}+\mathbf{Q}} \tag{16}$$

$$\mathscr{H}_{sd}^{(1)} = J_{sd}\sum_{\mathbf{k},\mathbf{q}} c_{\mathbf{k}}^{\dagger}\left(S_{\mathbf{q}}^{y}\sigma_1 + S_{\mathbf{q}}^{z}\sigma_2\right)c_{\mathbf{k}+\mathbf{q}} \tag{17}$$

The Hamiltonian is currently written in terms of spin operators, we want to move towards bosonic field operators, so we first do a Holstein-Primakoff transformation. The spin operators must obey the commutation relation

$$\left[S_i^y, S_j^z\right] = \mathrm{i}\delta_{ij}S_i^x = \mathrm{i}\delta_{ij}\mathrm{e}^{\mathrm{i}\mathbf{Q}\cdot\mathbf{R}_i}\left(S - \frac{(S_i^y)^2 + (S_i^z)^2}{2S}\right) \tag{18}$$

and we can write them in terms of Holstein-Primakoff operators (a_i, $a_i^{\dagger}$)

$$S_i^y \approx \sqrt{S}\frac{a_i + a_i^{\dagger}}{\sqrt{2}}, \quad S_i^z \approx \mathrm{e}^{\mathrm{i}\mathbf{Q}\cdot\mathbf{R}_i}\sqrt{S}\frac{a_i - a_i^{\dagger}}{\sqrt{2}\mathrm{i}} \tag{19}$$

The Fourier transformed operators are

$$S_{\mathbf{k}}^y = \sqrt{\frac{S}{2}}\left(a_{\mathbf{k}} + a_{-\mathbf{k}}^{\dagger}\right) \tag{20}$$

$$S_{\mathbf{k}}^z = -\mathrm{i}\sqrt{\frac{S}{2}}\left(a_{\mathbf{k}-\mathbf{Q}} - a_{-\mathbf{k}+\mathbf{Q}}^{\dagger}\right) \tag{21}$$

which after some work allows the AFM Hamiltonian to be written as

$$\mathscr{H}_{AFM} = S\sum_{\mathbf{k}}\begin{pmatrix}a_{\mathbf{k}}^{\dagger} & a_{-\mathbf{k}}\end{pmatrix}\begin{pmatrix}J_{\mathrm{AFM}}\gamma_0 + K & J_{\mathrm{AFM}}\gamma_{\mathbf{k}} \\ J_{\mathrm{AFM}}\gamma_{\mathbf{k}} & J_{\mathrm{AFM}}\gamma_0 + K\end{pmatrix}\begin{pmatrix}a_{\mathbf{k}} \\ a_{-\mathbf{k}}^{\dagger}\end{pmatrix} - \frac{H}{2}\sum_{\mathbf{k}}\left(a_{\mathbf{k}}^{\dagger}a_{\mathbf{k}+\mathbf{Q}} + a_{-\mathbf{k}}a_{-\mathbf{k}-\mathbf{Q}}^{\dagger}\right) \tag{22}$$

The first term here is the normal degenerate AFM spectrum, but the applied field breaks the translational symmetry. To calculate the eigenfrequencies and modes we rewrite as

$$\mathscr{H}_{\mathrm{AFM}} = \sum_{\mathbf{k}}{}' \begin{pmatrix}a_{\mathbf{k}}^{\dagger} & a_{-\mathbf{k}} & a_{\mathbf{k}+\mathbf{Q}}^{\dagger} & a_{-\mathbf{k}-\mathbf{Q}}\end{pmatrix}\begin{pmatrix}J_{\mathrm{AFM}}\gamma_0 + K & J_{\mathrm{AFM}}\gamma_{\mathbf{k}} & -H/2 & 0 \\ J_{\mathrm{AFM}}\gamma_{\mathbf{k}} & J_{\mathrm{AFM}}\gamma_0 + K & 0 & -H/2 \\ -H/2 & 0 & J_{\mathrm{AFM}}\gamma_0 + K & -J_{\mathrm{AFM}}\gamma_{\mathbf{k}} \\ 0 & -H/2 & -J_{\mathrm{AFM}}\gamma_{\mathbf{k}} & J_{\mathrm{AFM}}\gamma_0 + K\end{pmatrix}\begin{pmatrix}a_{\mathbf{k}} \\ a_{-\mathbf{k}}^{\dagger} \\ a_{\mathbf{k}+\mathbf{Q}} \\ a_{-\mathbf{k}-\mathbf{Q}}^{\dagger}\end{pmatrix} \tag{23}$$

where $\sum'$ means a sum over half the the Brillouin zone (i.e. not double counting the two AFM modes).

The s-d interaction can also be written in terms of Holstein-Primakoff operators which eventually gives

$$\mathscr{H}_{sd}^{(1)} = J_{sd}\sqrt{\frac{S}{2}}\sum_{\mathbf{k}}\sum_{\mathbf{q}}{}' \left[\left(a_{\mathbf{q}} - a_{\mathbf{q}-\mathbf{Q}} + a_{-\mathbf{q}}^{\dagger} + a_{-\mathbf{q}+\mathbf{Q}}^{\dagger}\right)c_{\mathbf{k}\uparrow}^{\dagger}c_{\mathbf{k}+\mathbf{q}\downarrow} - \left(a_{\mathbf{q}} - a_{\mathbf{q}+\mathbf{Q}} - a_{-\mathbf{q}}^{\dagger} - a_{-\mathbf{q}-\mathbf{Q}}^{\dagger}\right)c_{\mathbf{k}\uparrow}^{\dagger}c_{\mathbf{k}+\mathbf{q}+\mathbf{Q}\downarrow} + \mathrm{h.c.}\right] \tag{24}$$

We now diagonalise the AFM Hamiltonian and write everything in terms of bosonic eigenstates, $\alpha_{\mathbf{k}}^{\dagger}, \alpha_{\mathbf{k}}, \beta_{\mathbf{k}}^{\dagger}, \beta_{\mathbf{k}}$,which are creation and annihilation operators for each of the two AFM modes. This is done with a Bogoliubov-de Gennes transformation where the $u - v$ transform is

$$u_{\mathbf{k}} = \cosh\theta_{\mathbf{k},\pm}, \quad v_{\mathbf{k}} = \sinh\theta_{\mathbf{k}} \tag{25}$$

with

$$\tanh 2\theta_{\mathbf{k}} = -\frac{J_{\mathrm{AFM}}\gamma_{\mathbf{k}}}{J_{\mathrm{AFM}}\gamma_0 + K} \tag{26}$$

this allows us to write the Hamiltonians as

$$\mathscr{H}_{\mathrm{AFM}} = \sum_{\mathbf{k}}{}' \left[\left(\hbar\omega_{\mathbf{k}} - H\right) \alpha^{\dagger}_{\mathbf{k}}\alpha_{\mathbf{k}} + \left(\hbar\omega_{\mathbf{k}} + H\right) \beta^{\dagger}_{\mathbf{k}}\beta_{\mathbf{k}} \right] \tag{27}$$

and

$$\mathscr{H}_{sd} = \sqrt{\frac{S}{2}} \sum_{\mathbf{k}} \sum_{\mathbf{q}}{}' J_{sd} \left[(u_{\mathbf{q}} + v_{\mathbf{q}}) \left(\beta_{\mathbf{q}} + \alpha^{\dagger}_{-\mathbf{q}}\right) c^{\dagger}_{\mathbf{k}\uparrow} c_{\mathbf{k}+\mathbf{q}\downarrow} - (u_{\mathbf{q}} - v_{\mathbf{q}}) \left(\beta_{\mathbf{q}} - \alpha^{\dagger}_{-\mathbf{q}}\right) c^{\dagger}_{\mathbf{k}\uparrow} c_{\mathbf{k}+\mathbf{q}\downarrow} \right] + \mathrm{h.c.} \tag{28}$$

This is the sd Hamiltonian and AFM Hamiltonian which is the same as derived by others such as Chen et al[1].

II. INTERFACE SPIN CURRENT

Here we follow the work of Tveten *et al.*[2] which makes several assumptions:

- The sd coupling is not the dominant term in the Hamiltonian thus spin transfer from s to d can be treated in the mean-field with Fermi's Golden rule.
- All energy scales in the model are smaller than the Fermi energy ($k_{\mathrm{B}}T_{\mathrm{F}}$) so electron-hole pairs are mostly around the Fermi level.

First we simplify the magnon dispersion to make it easier to deal with. Essentially we are after an expression which is isotropic so that we can integrate over k rather than sum over $\mathbf{k}$. This makes a tacit assumption that the shape of the Brillouin zone at the edges is not important. We aim for something of the form $\varepsilon_{\mathbf{k}} = \varepsilon_0 + Ak$.

The full expression for the magnon dispersion is

$$\varepsilon_{\mathbf{k}} = \hbar\omega_{\mathbf{k}} = 2S\sqrt{(J_{\mathrm{AFM}}\gamma_0 + K)^2 - J^2_{\mathrm{AFM}}\gamma^2_{\mathbf{k}}} \mp g\mu_B\mu_0 H_{\mathrm{ext}} \tag{29}$$

We simplify this by assuming $J_{\mathrm{AFM}} \gg K$ and $J_{\mathrm{AFM}} \gg g\mu_B\mu_0 H_{\mathrm{ext}}$. The dispersive part is then

$$\varepsilon_{\mathbf{k}} \approx 4\sqrt{3}SJ_{\mathrm{AFM}}k \tag{30}$$

and the gap is

$$\varepsilon_0 \approx 2S\sqrt{(J_{\mathrm{AFM}}z + K)K} \mp g\mu_B\mu_0 H_{\mathrm{ext}} \tag{31}$$

Hence our approximate dispersion is

$$\varepsilon_{\mathbf{k}} \approx 4\sqrt{3}SJ_{\mathrm{AFM}}k + 2S\sqrt{(J_{\mathrm{AFM}}z + K)K} \mp g\mu_B\mu_0 H_{\mathrm{ext}} \tag{32}$$

For the case of $KCoF_3$ the true dispersion is quite curved at low k vectors (due to the relatively high K) and so the approximation is not so good there.

Now that we have a simple expression we can easily derive the density of magnon states in the AFM

$$g(E)=\frac{d\Omega(E)}{dE}=\frac{4\pi}{A^3}(\varepsilon_k-\varepsilon_0)^2=\frac{4\pi k^2}{A} \tag{33}$$

The rate of spin transfer from s to d systems comes from Fermi's Golden rule. Again, following Tveten et al.[2] we assume dephasing effects are large enough that we can define the density matrix as the product $\hat{\rho}_{sd}=\hat{\rho}_s\otimes\hat{\rho}_d$ allowing us to take the partial trace when we do Fermi's Golden rule.

The spin current due to sd scatting by Fermi's Golden rule is

$$\begin{aligned}
I_{\text{sd}}=\frac{\pi S J_{sd}^2}{A}\sum_{\mathbf{k}}\sum_{\mathbf{q}}{}' \Bigg[&\operatorname{Tr}\left\{(u_{\mathbf{q}}+v_{\mathbf{q}})^2\alpha_{-\mathbf{q}}c^\dagger_{\mathbf{k+q},\downarrow}c_{\mathbf{k},\uparrow}\hat{\rho}_{\text{sd}}\alpha^\dagger_{-\mathbf{q}}c^\dagger_{\mathbf{k},\uparrow}c_{\mathbf{k+q},\downarrow}\right\}\delta(\varepsilon_{\mathbf{k}}+\varepsilon^\alpha_{-\mathbf{q}}-\varepsilon_{\mathbf{k+q}})\\
&-\operatorname{Tr}\left\{(u_{\mathbf{q}}+v_{\mathbf{q}})^2\alpha^\dagger_{-\mathbf{q}}c^\dagger_{\mathbf{k},\uparrow}c_{\mathbf{k+q},\downarrow}\hat{\rho}_{\text{sd}}\alpha_{-\mathbf{q}}c^\dagger_{\mathbf{k+q},\downarrow}c_{\mathbf{k},\uparrow}\right\}\delta(\varepsilon_{\mathbf{k}}+\varepsilon^\alpha_{-\mathbf{q}}-\varepsilon_{\mathbf{k+q}})\\
&+\operatorname{Tr}\left\{(u_{\mathbf{q}}+v_{\mathbf{q}})^2\beta^\dagger_{\mathbf{q}}c^\dagger_{\mathbf{k+q},\downarrow}c_{\mathbf{k},\uparrow}\hat{\rho}_{\text{sd}}\beta_{\mathbf{q}}c^\dagger_{\mathbf{k},\uparrow}c_{\mathbf{k+q},\downarrow}\right\}\delta(\varepsilon_{\mathbf{k}}-\varepsilon^\beta_{\mathbf{q}}-\varepsilon_{\mathbf{k+q}})\\
&-\operatorname{Tr}\left\{(u_{\mathbf{q}}+v_{\mathbf{q}})^2\beta_{\mathbf{q}}c^\dagger_{\mathbf{k},\uparrow}c_{\mathbf{k+q},\downarrow}\hat{\rho}_{\text{sd}}\beta^\dagger_{\mathbf{q}}c^\dagger_{\mathbf{k+q},\downarrow}c_{\mathbf{k},\uparrow}\right\}\delta(\varepsilon_{\mathbf{k}}-\varepsilon^\beta_{\mathbf{q}}-\varepsilon_{\mathbf{k+q}})\\
&+\operatorname{Tr}\left\{(u_{\mathbf{q}}-v_{\mathbf{q}})^2\alpha_{-\mathbf{q}}c^\dagger_{\mathbf{k+q+Q},\downarrow}c_{\mathbf{k},\uparrow}\hat{\rho}_{\text{sd}}\alpha^\dagger_{-\mathbf{q}}c^\dagger_{\mathbf{k},\uparrow}c_{\mathbf{k+q+Q},\downarrow}\right\}\delta(\varepsilon_{\mathbf{k}}+\varepsilon^\alpha_{-\mathbf{q}}-\varepsilon_{\mathbf{k+q+Q}})\\
&-\operatorname{Tr}\left\{(u_{\mathbf{q}}-v_{\mathbf{q}})^2\alpha^\dagger_{-\mathbf{q}}c^\dagger_{\mathbf{k},\uparrow}c_{\mathbf{k+q+Q},\downarrow}\hat{\rho}_{\text{sd}}\alpha_{-\mathbf{q}}c^\dagger_{\mathbf{k+q+Q},\downarrow}c_{\mathbf{k},\uparrow}\right\}\delta(\varepsilon_{\mathbf{k}}+\varepsilon^\alpha_{-\mathbf{q}}-\varepsilon_{\mathbf{k+q+Q}})\\
&+\operatorname{Tr}\left\{(u_{\mathbf{q}}-v_{\mathbf{q}})^2\beta^\dagger_{\mathbf{q}}c^\dagger_{\mathbf{k+q+Q},\downarrow}c_{\mathbf{k},\uparrow}\hat{\rho}_{\text{sd}}\beta_{\mathbf{q}}c^\dagger_{\mathbf{k},\uparrow}c_{\mathbf{k+q+Q},\downarrow}\right\}\delta(\varepsilon_{\mathbf{k}}-\varepsilon^\beta_{\mathbf{q}}-\varepsilon_{\mathbf{k+q+Q}})\\
&-\operatorname{Tr}\left\{(u_{\mathbf{q}}-v_{\mathbf{q}})^2\beta_{\mathbf{q}}c^\dagger_{\mathbf{k},\uparrow}c_{\mathbf{k+q+Q},\downarrow}\hat{\rho}_{\text{sd}}\beta^\dagger_{\mathbf{q}}c^\dagger_{\mathbf{k+q+Q},\downarrow}c_{\mathbf{k},\uparrow}\right\}\delta(\varepsilon_{\mathbf{k}}-\varepsilon^\beta_{\mathbf{q}}-\varepsilon_{\mathbf{k+q+Q}})\Bigg]
\end{aligned} \tag{34}$$

where the delta functions enforce conservation of momentum and the sign of each term is due to the direction of the spin current where we define angular momentum transfer which changes the electron's spin $\uparrow$ to $\downarrow$ as positive.

The expectation value of the number operators will give us the distribution functions as $\langle A\rangle=\operatorname{Tr}(\rho A)$ hence

$$\langle c^\dagger_{k\sigma}c_{k'\sigma'}\rangle=\operatorname{Tr}(\hat{\rho}_{\text{s}}c^\dagger_{k\sigma}c_{k'\sigma'})=n_F(\varepsilon_k-\mu_\sigma)\delta_{kk'}\delta_{\sigma\sigma'} \tag{35}$$

where $n_{\text{FD}}(\varepsilon_k-\mu_\sigma)$ is the Fermi-Dirac distribution for the electrons with chemical potential μ_σ and we assume they thermalise quickly to this distribution.

The magnons are not assumed to be in thermal equilibrium and take a general distribution function which is distinct for both branches

$$\langle\alpha^\dagger_q\alpha_{q'}\rangle=\operatorname{Tr}(\hat{\rho}_{\text{s}}\alpha^\dagger_q\alpha_{q'})=n_\alpha(\varepsilon^\alpha_q)\delta_{qq'} \tag{36}$$

$$\langle\beta^\dagger_q\beta q'\rangle=\operatorname{Tr}(\hat{\rho}_{\text{s}}\beta^\dagger_q\beta_{q'})=n_\beta(\varepsilon^\beta_q)\delta_{qq'} \tag{37}$$

Working through some tedious maths and using the identities $n_F(x)[1-n_F(y)]=n_B(x-y)[n_F(y)-n_F(x)]$ and $1+n_B(x)=$

$-n_B(-x)$ and $\mu_s = \mu_\uparrow - \mu_\downarrow$ and the fact the dispersion obeys $\varepsilon^\alpha_{-\mathbf{q}} = \varepsilon^\alpha_{\mathbf{q}}$. We get to

$$
\begin{aligned}
I_{\rm sd} = \frac{\pi S J_{sd}^2}{A} \sum_{\mathbf{k},\mathbf{k}'} \sum_{\mathbf{q}}{}' \left[(u_\mathbf{q}+v_\mathbf{q})^2 + (u_\mathbf{q}-v_\mathbf{q})^2\right] \Bigg\{ \\
&\Bigg[[1+n_\alpha(\varepsilon^\alpha_\mathbf{q})] n_B(\varepsilon_{\mathbf{k}'} - \varepsilon_\mathbf{k} - \mu_\downarrow + \mu_\uparrow)[n_F(\varepsilon_\mathbf{k} - \mu_\uparrow) - n_F(\varepsilon_{\mathbf{k}'} - \mu_\downarrow)] \\
&- [n_\alpha(\varepsilon^\alpha_\mathbf{q})] n_B(\varepsilon_\mathbf{k} - \varepsilon_{\mathbf{k}'} + \mu_\downarrow - \mu_\uparrow)[n_F(\varepsilon_{\mathbf{k}'} - \mu_\downarrow) - n_F(\varepsilon_\mathbf{k} - \mu_\uparrow)] \Bigg] \delta(\varepsilon_\mathbf{k} + \varepsilon^\alpha_\mathbf{q} - \varepsilon_{\mathbf{k}'}) \\
&\Bigg[[1+n_\beta(\varepsilon^\beta_\mathbf{q})] n_B(\varepsilon_\mathbf{k} - \varepsilon_{\mathbf{k}'} + \mu_\downarrow - \mu_\uparrow)[n_F(\varepsilon_{\mathbf{k}'} - \mu_\downarrow) - n_F(\varepsilon_\mathbf{k} - \mu_\uparrow)] \\
&- [n_\beta(\varepsilon^\beta_\mathbf{q})] n_B(\varepsilon_{\mathbf{k}'} - \varepsilon_\mathbf{k} - \mu_\downarrow + \mu_\uparrow)[n_F(\varepsilon_\mathbf{k} - \mu_\uparrow) - n_F(\varepsilon_{\mathbf{k}'} - \mu_\downarrow)] \Bigg] \delta(\varepsilon_\mathbf{k} - \varepsilon^\beta_\mathbf{q} - \varepsilon_{\mathbf{k}'}) \Bigg\}
\end{aligned}
\tag{38}
$$

We assume a continuous density of states and change from summations over wave vectors to integrals over energy. We further assume that s-electron-hole pairs are all distributed close to the Fermi level so $g_\uparrow(\varepsilon) \approx g_\downarrow(\varepsilon) \approx g(E_F) = D$.

Evaluating the integrals over $\varepsilon_\mathbf{k}$ and $\varepsilon_{\mathbf{k}'}$ and using the identities $n_B(-E) = -(1+n_B(E))$ and $\int_{-\infty}^{\infty} [n_F(\varepsilon) - n_F(\varepsilon+\omega)] = \omega$ and taking the area per unit cell $A = a^2_{\rm NM}$ brings us to the result

$$
\begin{aligned}
I_{\rm sd} = \pi S J_{sd}^2 D^2 a^4_{\rm NM} a^3_{\rm AF} \Bigg\{ &\int_{\varepsilon_0^\alpha}^{\varepsilon^\alpha_{\rm max}} d\varepsilon^\alpha_\mathbf{q} g_\alpha(\varepsilon^\alpha_\mathbf{q}) \left[(u_\mathbf{q}+v_\mathbf{q})^2 + (u_\mathbf{q}-v_\mathbf{q})^2\right] [\varepsilon^\alpha_\mathbf{q} + \mu_s][n_B(\varepsilon^\alpha_\mathbf{q} + \mu_s) - n_\alpha(\varepsilon^\alpha_\mathbf{q})] \\
&- \int_{\varepsilon_0^\beta}^{\varepsilon^\beta_{\rm max}} d\varepsilon^\beta_\mathbf{q} g_\beta(\varepsilon^\beta_\mathbf{q}) \left[(u_\mathbf{q}+v_\mathbf{q})^2 + (u_\mathbf{q}-v_\mathbf{q})^2\right] [\varepsilon^\beta_\mathbf{q} - \mu_s][n_B(\varepsilon^\beta_\mathbf{q} - \mu_s) - n_\beta(\varepsilon^\beta_\mathbf{q})] \Bigg\}
\end{aligned}
\tag{39}
$$

where $\varepsilon_0^{\alpha,\beta}$ and $\varepsilon^{\alpha,\beta}_{\rm max}$ are the minimum and maximum energies of the two AFM bands at the zone center and zone edge respectively. $g_\beta(\varepsilon^{\alpha,\beta}_\mathbf{q})$ is the magnon density of states of each band.

III. SPIN ACCUMULATION DYNAMICS

Now we have an expression for the spin current we need an equation of motion for how the spin accumulation in the metal and the non-equilibrium magnon distribution in the AFM evolve. Here we follow very directly Tveten's work[2]. Within the already stated assumptions of the electron-hole pairs being distributed close the the Fermi level the spin accumulation is

$$
\mu_s = \delta\mu_\uparrow - \delta\mu_\downarrow = \frac{\delta n_s}{D} + \delta\Delta_{xc} \tag{40}
$$

where δn_s is the out-of-equilibrium spin density, $D = 2D^{\varepsilon_F}_\uparrow D^{\varepsilon_F}_\downarrow / (D^{\varepsilon_F}_\uparrow + D^{\varepsilon_F}_\downarrow)$ and $\delta\Delta_{xc} = \delta\Delta_\uparrow - \delta\Delta_\downarrow$ is the non-equilibrium change in exchange splitting due to the magnetisation dynamics. In the ultrafast regime we can ignore the second term. The equation of motion for the spin accumulation is then

$$
\frac{\partial \mu_s}{\partial t} = -\frac{\mu_s}{\tau_s} + \frac{\rho}{\hbar} I_{sd} \tag{41}
$$

where τ_s is the relaxation time of s-electron spins in the Pt and $\rho = -1/D$. Negative means the s-d coupling Hamiltonian is ferromagnetic in nature.

The general magnon distribution functions evolve in time as

$$
\frac{\partial n_\alpha(\varepsilon^\alpha_q)}{\partial t} = \frac{I_{sd}(\varepsilon^\alpha_q)}{\hbar} = \frac{1}{\hbar} \pi S J^2_{sd} D^2 a^4_{\rm NM} a^3_{\rm AF} g_\alpha(\varepsilon^\alpha_q) \left[(u_q+v_q)^2 + (u_q-v_q)^2\right] [\varepsilon^\alpha_q + \mu_s][n_B(\varepsilon^\alpha_q + \mu_s) - n_\alpha(\varepsilon^\alpha_q)] \tag{42}
$$

$$\frac{\partial n_\beta(\varepsilon_q^\beta)}{\partial t} = \frac{I_{sd}(\varepsilon_q^\beta)}{\hbar} = -\frac{1}{\hbar}\pi S J_{sd}^2 D^2 a_{\rm NM}^4 a_{\rm AF}^3 g_\beta(\varepsilon_q^\beta)\left[(u_q+v_q)^2+(u_q-v_q)^2\right][\varepsilon_q^\beta-\mu_s][n_B(\varepsilon_q^\beta-\mu_s)-n_\beta(\varepsilon_q^\beta)] \quad (43)$$

This assumes that there is no direct scattering between magnons, either from different branches (α, β) or different q points in the timescale of a few picosceonds.

The temperature comes in through the Bose-Einstein distribution $n_B(x)$ and we use a simple model for the evolution of the electronic temperature due to the laser heating

$$\frac{\partial T_e}{\partial t} = \frac{T_0 - T_e}{\tau_e} + I\mathrm{e}^{-\frac{(t-t_0)^2}{2\sigma^2}} \quad (44)$$

where T_e is the temperature of the electrons in the Pt, T_0 is the initial temperature, τ_e is a decay time for the electronic temperature, I is a measure of the laser power, t_0 is the temporal centre of the laser pulse and σ is the temporal width of the laser pulse.

IV. MAGNETIC DOMAINS IN $KCoF_3$ AND $KNiF_3$

Fig.1(a) shows susceptibility measurements performed by SQUID (Superconducting QUantum Interference Device) magnetometry in $KCoF_3$ and $KNiF_3$ as a function of temperature and for zero and 1 T cooling field along the [001] crystal axis. The magnetic susceptibility of $KCoF_3$ has a much weaker temperature dependence below T_N than $KNiF_3$. The unusual susceptibility curve of $KCoF_3$ has been known since the 70's[4–6]. In these works the high value of the longitudinal susceptibility at low temperatures is attributed to the residual magnetic moment originating in the unquenched orbital angular momentum of the Co^{2+} atoms. Moreover, strain is responsible for domain rearrangement, favouring those domains with the Néel vector parallel to the stress direction. Our susceptibility curve shown in Fig.1 of the main text is very similar to what measured in Fig.5 of [5] when the $KCoF_3$ specimen is subjected to a perpendicular strain. In our measurements, we don't actively apply a stress to our sample, but we think that strain might originate from the way the sample is mounted and glued. Although we have not carried a systematic study on strain, we have observed that magnetic susceptibility curves measured on the same $KCoF_3$ specimen can vary significantly, not in the same measurement session, but when the sample is taken out and remounted. We provide an example in Fig.1(b). Here the susceptibility is measured along the [001] crystal direction and no magnetic field is applied during cool down. The higher susceptibility in run 1 suggests a larger contribution from the domains with the Néel vector oriented in the (001) plane.

In Fig.1(a) the increase of susceptibility below T_N when a magnetic field is applied during cool down is explained with the expansion of domains with the Néel vector perpendicular to the applied field. The critical field for domain wall motion can be extracted from the SQUID measurements presented in Fig. 1(c) and (d). From these graphs we can see that the net magnetic moment is characterised by a non-linear increase for the external field exceeding a critical value of $\sim 0.5\ T$ in $KNiF_3$ and $\sim 2.9\ T$ in $KCoF_3$. This increase is explained with the expansion of domains with the Néel vector perpendicular to the field, at the expenses of those with the Néel vector parallel to it, and the critical field values are in agreement with those reported in a previous study[7] for both materials. In Fig. 1(d) we also notice that the magnetic curves in $KCoF_3$ are characterised by a small hysteresis. We associate this to a small magnetic component that survives above T_N. This is also reflected in our THz emission measurements, which show a small hysteresis at low magnetic field. The exact origin of the net moment is unclear. It might originate from microscopic Co islands existing in the sample, however any effect connected to ferromagnetism is easily separable from those originating in the antiferromagent, which decay at T_N.

For the THz emission measurements presented in the main text, the maximum field we can apply is 0.85 T. Although this is above the critical field for irreversible domain wall motion in $KNiF_3$ it is considerably lower than this critical value for $KCoF_3$. So, while in $KNiF_3$ we can reasonably assume that mainly two domains, the [010] and the [001] contribute to the THz emission, in $KCoF_3$ we have to consider a contribution from all of the domains, [100], [010] and [001]. In the small oscillation limit both $KNiF_3$ and $KCoF_3$ can be approximated to uniaxial antiferromagnets, in which the k=0 magnon modes are characterised by the same energy at zero field for both types of domains, with the Néel vector parallel or perpendicular to the external magnetic field. This was also confirmed in the case of $KNiF_3$ by far-infrared absorption spectroscopy[3]. In our work we explain the attenuation in the THz emission below T_N with the opening of the gap at the centre of the magnon Brillouin zone. Because all three types of domains are characterised by the same magnitude of the gap, we expect a similar attenuation of the picosecond spin-Seebeck effect. For domains not aligned with the field there may also be a contribution to the spin current from coherent spin torques[8] and the non-linear spin-Seebeck effect[9]. The coherent spin torques on each sublattice cancel due to symmetry and because the distribution of up and down electrons in the Pt is not significantly altered. The non-linear SSE will contribute proportional to the induced magnetisation which is extremely small at these fields.

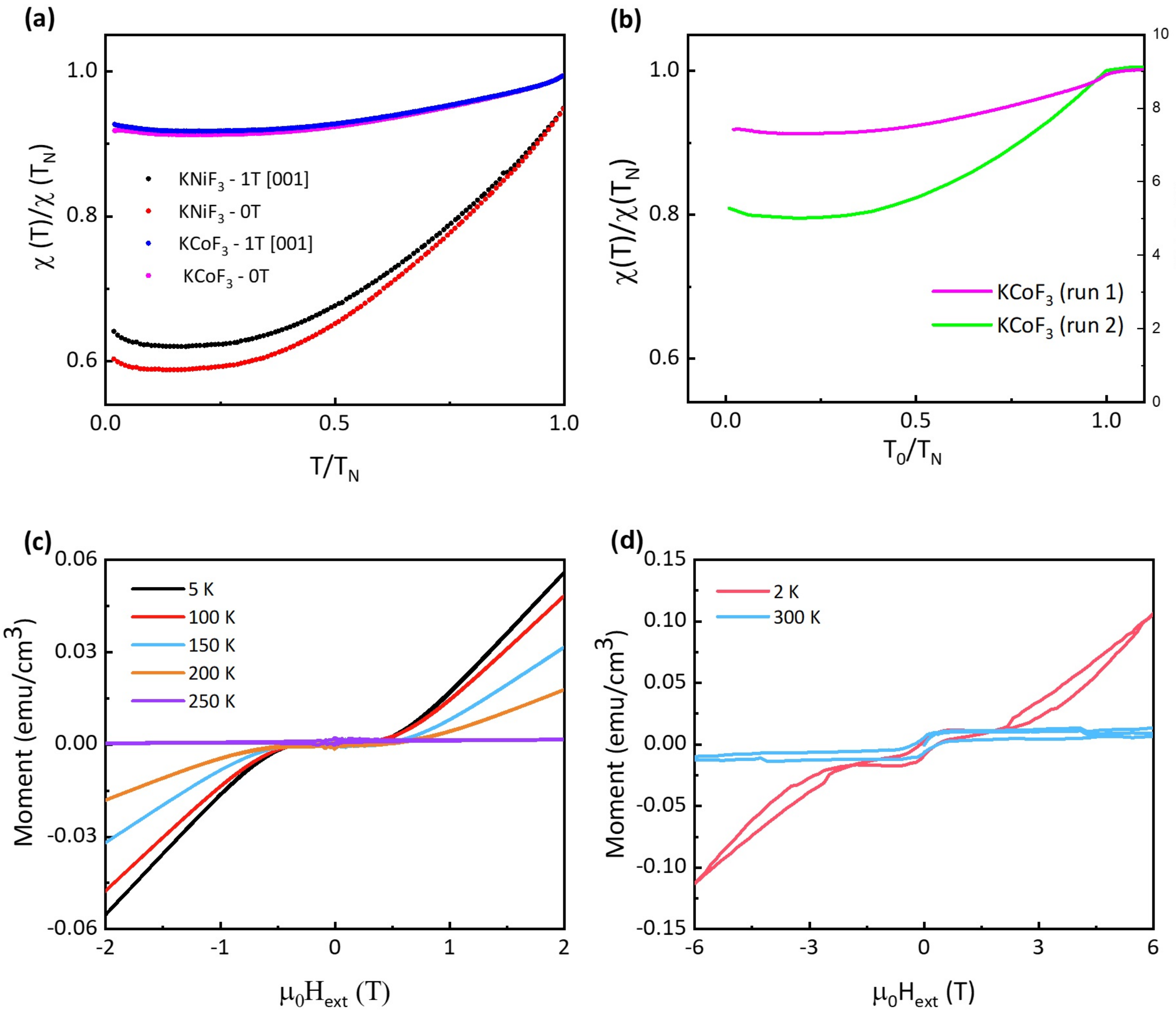

FIG. 1. (a) Normalised susceptibility in $KNiF_3$ (b) and $KCoF_3$ as a function of the normalised temperature. The measurement was done in both cases at zero magnetic field and for a 1 T magnetic field applied in the [001] crystal direction. (b) Normalised susceptibility measured in two different SQUID sessions in KCoF3. (c)-(d) M-H plot of $KNiF_3$ (b) and $KCoF_3$ (c) measured at different temperatures after subtracting a linear background.

V. PUMP-PROBE MEASUREMENT OF THE K=0 MAGNON MODE FREQUENCY AS A FUNCTION OF TEMPERATURE

Here we reproduce the measurements of the magnon frequency performed in $KCoF_3$ and $KNiF_3$ and published elsewhere[14]. A diagram of the measurement scheme is shown in Fig. 2(a). A 1.5 eV pump pulse with fluence of 25 mJ/cm^2 was used to excite coherent oscillations via impulsive stimulated Raman scattering. A delayed 3 eV linearly polarised probe, generated by frequency-doubling the fundamental beam with a BBO crystal, was used to probe the excited spin dynamics by detecting changes in the probe polarization plane. A magnetic field was applied along the direction of propagation of the pump beam, so the spin dynamics were measured in the Faraday geometry. The frequency of the oscillations as a function of temperature is shown in Fig. 2(b) and (c) and gives a direct measure of the magnon gap in the two materials. As expected the gap closes as we approach the Néel temperature.

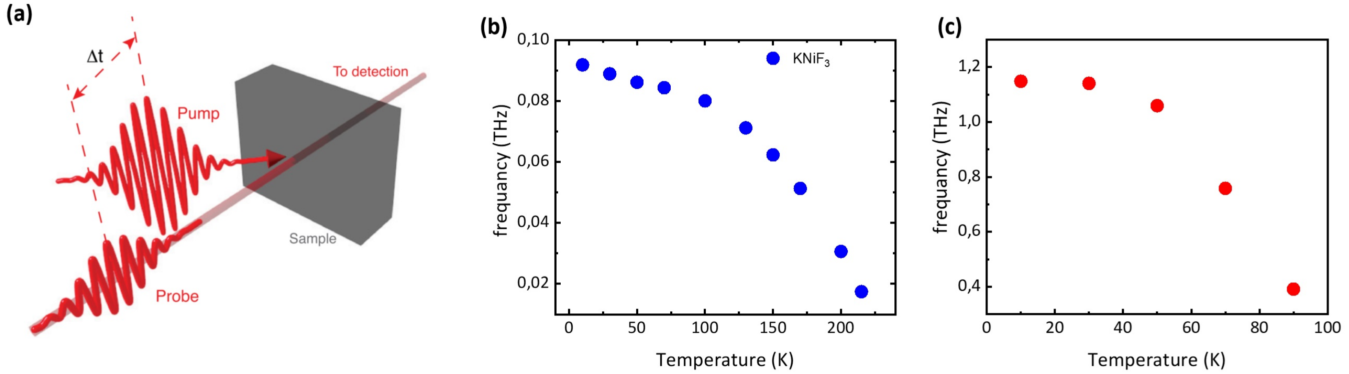

FIG. 2. (a) Schematics of the measurement set-up. (b)-(c) Temperature dependence of the frequency of the coherent oscillations in $KNiF_3$ and $KCoF_3$ as a function of temperature.

VI. THZ EMISSION IN THE PARAMAGNETIC PHASE OF KCOF$_3$ (T> T$_N$)

In Fig.3 (a) we show that the emitted THz does not depend on the polarisation of the pump pulse, hinting at its thermal origin. The THz pulse is polarised in the direction perpendicular to the external magnetic field and its amplitude scales linearly with it (Fig.3(b)), in agreement with a spin-Seebeck picture. In the paramagnetic phase the spin Seebeck signal cannot be interpreted in terms of magnons since there is no long-range magnetism, but also in the absence of a spontaneous magnetic moment the magnetic field can induce a finite magnetic moment proportional to the magnetic susceptibility. In this case the temperature dependence of the spin Seebeck effect can be entirely described in terms of the critical scaling of the susceptibility as a function of two parameters: the reduced temperature $t = (T - T_N)/T_N$ and the magnetic field[10]. Differently from what observed in $KCoF_3$ below T_N (Fig. 3a of the main article), in this case the fluence of the pump does not change the scaling law as it has no impact on magnetic ordering or the susceptibility. As expected, Fig. 3(c) shows that the temperature dependence of the picosecond spin Seebeck signal in $KCoF_3$ does not depend on fluence at $T > T_N$. Moreover, this temperature dependence overlaps with the temperature dependence of the spin Seebeck effect measured in the quasi-dc limit and in a completely different antiferromagnet, FeF_2[11].

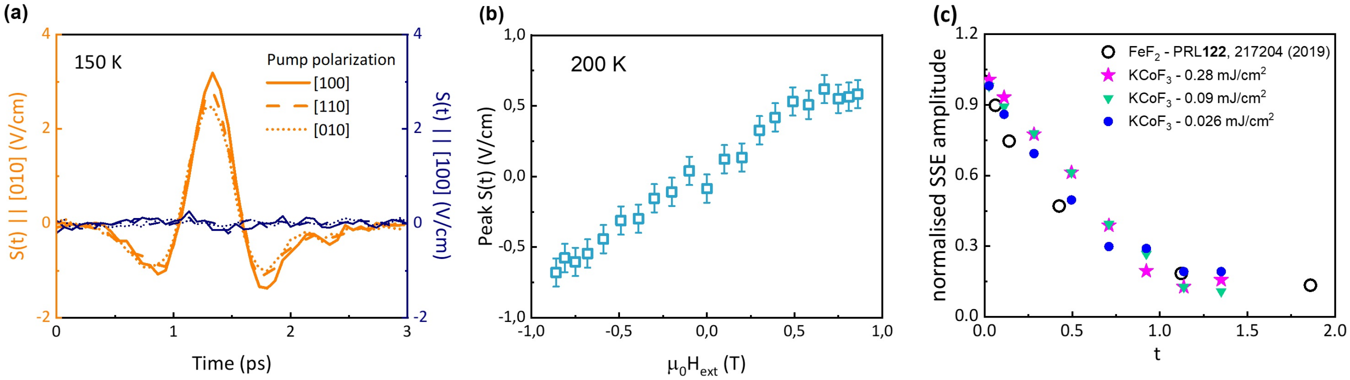

FIG. 3. (a) Pump polarization dependence of the THz time-domain trace from $KCoF_3/Pt$ at $T = 150\ K$, polarised along the [010] direction, perpendicular to the external magnetic field, and along the [100] direction, parallel to the external magnetic field. (b) Field dependence of the peak THz field emission measured at $T = 200\ K$. (c) Normalised spin-Seebeck amplitude as a function of the reduced temperature above the Néel temperature.

VII. UNNORMALISED AMPLITUDE OF THE THZ EMISSION AS A FUNCTION OF TEMPERATURE

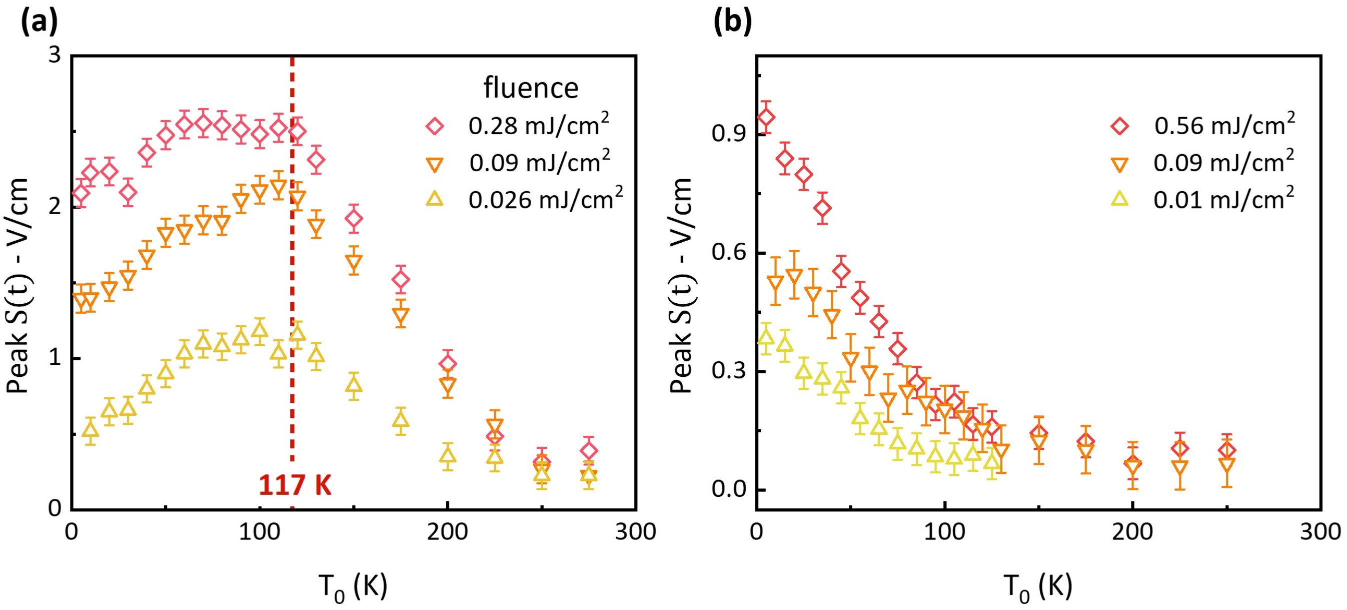

FIG. 4. Absolute amplitude of the THz electric field pulse emitted by $KCoF_3$ (a) and $KNiF_3$ (b) as a function of ambient temperature and for different fluences.

VIII. COMPARISON BETWEEN THE ABSOLUTE MAGNITUDES OF THE PICOSECOND SPIN-SEEBECK EFFECT IN $KNiF_3/Pt$, $KCoF_3/Pt$ AND YIG/Pt

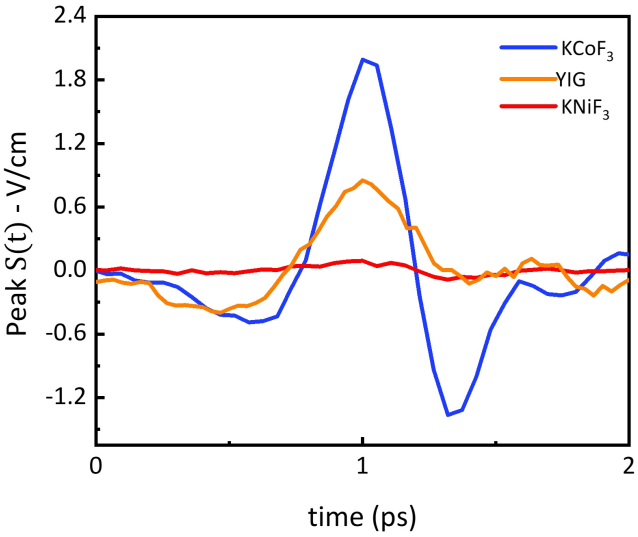

FIG. 5. Time traces of the emitted THz pulse from KCoF3, KNiF3 and YIG, measured at the same pump fluence and at ambient temperature of 120K.

In Fig. 5 we compare the absolute amplitude of the emitted THz pulse in $KNiF_3/Pt$, $KCoF_3/Pt$ and YIG/Pt, measured for the same pump fluence and at the same ambient temperature of 120 K. According to theory[12] the paramagnetic spin Seebeck effect and the antiferromagnetic spin Seebeck effect at elevated temperatures above T_N are expressed by a single equation that is proportional to the external magnetic field times the spin susceptibility of the magnet. We therefore expect the amplitude

of the THz emission to be more than ten times larger in $KCoF_3$ than in $KNiF_3$ at their respective Néel temperatures. The proportionality of the spin-Seebeck effect on susceptibility is no longer valid below T_N. Even if for YIG and small gap antiferromagnets like $KNiF_3$ and MnF_2[13] the temperature dependence of the spin-Seebeck effect has a critical-like behaviour described by the power low $[(T_N - T)/T_N]^3$ and does not depend on pump fluence, to the best of our knowledge no satisfactory explanation has been given for the exponent 3, which is not related to the critical exponent for the magnetization, nor for the magnetic susceptibility. So, it is not surprising that, despite YIG has orders of magnitude larger magnetic moment at 1 T in comparison to the antiferromagnets, the amplitude of the spin-Seebeck effect in YIG and $KCoF_3$ are comparable, further confirming that it's not the magnetic susceptibility that plays the key role. Further studies from the theoretical and experimental point of view are desirable.

IX. THZ EMISSION FOR THE SINGLE KCOF$_3$ LAYER AND SINGLE PT LAYER

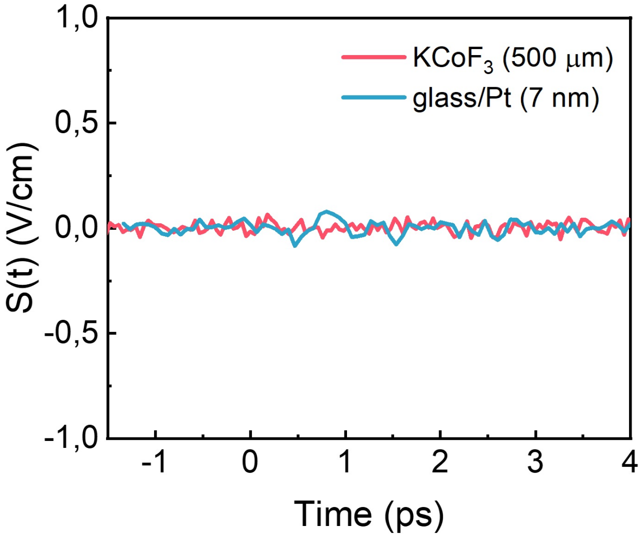

FIG. 6. This constitutes a check measurement. In order to observe Thz emission both the antiferromagnet, as spin source, and the Pt, as spin-to-charge transducer, are necessary.

[1] K. Chen, W. Lin, C.L. Chien and S. Zhang, "Temperature dependence of angular momentum transport across interfaces," *Phys. Rev. B*, **94**, 054413, (1999).

[2] E.G. Tveten, A. Brataas and Y. Tserkovnyak, "Electron-magnon scattering in magnetic heterostructures far out of equilibrium," *Phys. Rev. B*, **92**, 180412 (2015).

[3] Yamaguchi, H., Katsumata, K., Hagiwara, M., Tokunaga, M., Liu, H. L., Zibold, A., Tanner, D. B. and Wang, Y. J., "Antiferromagnetic resonance in the cubic perovskite $KNiF_3$," *Phys. Rev. B*, **59**, 6021–6023, (1999).

[4] K. Hirakawa and K. Hirakawa and T. Hashimoto, "Magnetic Properties of Potassium Iron Group Fluorides KMF3," *Journal of the Physical Society of Japan*, **15**, 2063, (1960).

[5] T. Tsuda and H. Yasuoka and T. Miyauchi, "Magnetic Susceptibility of an Antiferromagnetic KCoF3 Single Crystal–19F NMR and Static Measurements,," *Journal of the Physical Society of Japan*, **45**, 1551 , (1978).

[6] N. Suzuki and T. Isu and K. Motizuki, "Theoretical study of magnetic susceptibility of orbitally unquenched compound KCoF3,," *Solid State Communications* , **23**, 319 , (1977).

[7] M. Safa and B. K. Tanner, "Antiferromagnetic domain wall motion in KNiF3 and KCoF3 observed by X-ray synchrotron topography," *Philosophical Magazine B*, **37**, 739–750, (1978).

[8] R. Cheng and J. Xiao and Q. Niu and A. Brataas, "Spin Pumping and Spin-Transfer Torques in Antiferromagnets," *Physical Review Letters*, **113**, 7057601, (1914).

[9] T.S. Seifert and S. Jaiswal and J. Barker et al., "Femtosecond formation dynamics of the spin Seebeck effect revealed by terahertz spectroscopy," *Nature Communications*, **9**, 2899, (1918).

[10] M.D.Vannette and A.S.Sefat and S.Jia and S.A.Law and G.Lapertot and S.L.Bud'ko and P.C.Canfield and J.Schmalian and R.Prozorov, "Precise measurements of radio-frequency magnetic susceptibility in ferromagnetic and antiferromagnetic materials," *Journal of Magnetism and Magnetic Materials* , **320**, 354, (2008).

[11] J. Li and Z. Shi and V. Ortiz and M. Aldosary and C. Chen and V. Aji and P. Wei and J. Shi, "Spin Seebeck Effect from Antiferromagnetic Magnons and Critical Spin Fluctuations in Epitaxial FeF2 Films," *Physical Reviw Letters*, **122**, 217204 , (2019).

[12] Y. Yamamoto and M. Ichioka and H. Adachi, "Spin Seebeck effect in paramagnets and antiferromagnets at elevated temperatures," *Physical Reviw B*, **100**, 064419 , (2019).

[13] S.M. Wu and W. Zhang and Amit KC and P. Borisov and J.E. Pearson and J.S. Jiang and D. Lederman and A. Hoffmann, and A. Bhattacharya, "Antiferromagentic spin Seebeck effect," *Physical Reviw Letters*, **116**, 097204 , (2016).

[14] D. Bossini , "Femtosecond Optical Excitation of Spins in Antiferromagnetic fluorides," *Ipskamp Drukkers Enschede*, The Netherlands, (2015).
